# 292. Risk factor and clinical characteristics of pneumothorax and pneumomediastinum in COVID-19: a matched case-control study

**DOI:** 10.1093/ofid/ofac492.370

**Published:** 2022-12-15

**Authors:** Se Ju Lee, Jinnam Kim, Ki Hyun Lee, Jung Ah Lee, Chang Hyup Kim, Su Hwan Lee, Joon-sup Yeom, Nam Su Ku, Su Jin Jeong, Jin Young Ahn, Jung Ho Kim, Jun Yong Choi

**Affiliations:** Inha University College of Medicine, Seoul, Seoul-t'ukpyolsi, Republic of Korea; Yonsei University College of Medicine, Seoul, Seoul-t'ukpyolsi, Republic of Korea; Yonsei University College of Medicine, Seoul, Seoul-t'ukpyolsi, Republic of Korea; Yonsei University College of Medicine, Seoul, Seoul-t'ukpyolsi, Republic of Korea; Yonsei University College of Medicine, Seoul, Seoul-t'ukpyolsi, Republic of Korea; Yonsei University College of Medicine, Seoul, Seoul-t'ukpyolsi, Republic of Korea; Division of Infectious Diseases, Department of Internal Medicine, Yonsei University College of Medicine, Seoul, Seoul-t'ukpyolsi, Republic of Korea; Division of Infectious Diseases, Department of Internal Medicine, Yonsei University College of Medicine, Seoul, Seoul-t'ukpyolsi, Republic of Korea; Yonsei University College of Medicine, Seoul, Seoul-t'ukpyolsi, Republic of Korea; Yonsei University College of Medicine, Seoul, Seoul-t'ukpyolsi, Republic of Korea; Yonsei University College of Medicine, Seoul, Seoul-t'ukpyolsi, Republic of Korea; Yonsei University College of Medicine, Seoul, Seoul-t'ukpyolsi, Republic of Korea

## Abstract

**Background:**

During the novel coronavirus SARS-CoV-2 pandemic, a considerable number of pneumothorax and pneumomediastinum associated with COVID-19 have been reported, and the incidence was higher in critically ill patients. Despite using a protective ventilation strategy, barotrauma still occurs in COVID-19 patients with invasive mechanical ventilation. This study aims to identify the risk factors and clinical characteristics of pneumothorax and pneumomediastinum in COVID-19 by a matched case-control study.

**Methods:**

This retrospective study enrolled adult patients diagnosed with a COVID-19, admitted to a critical care unit in South Korea from 2020 March 1st to 2022 January 31st. COVID-19 patients with pneumothorax and pneumomediastinum were compared, in a 1 to 2 ratio, to a control group of COVID-19 patients without pneumothorax and pneumomediastinum, matched on age, gender, and worst National Institute of Allergy and Infectious Diseases ordinal scale (NIAID-OS). Conditional logistic regression analysis was performed to assess the risk factors for pneumothorax and pneumomediastinum in COVID-19.

**Results:**

A total of 427 patients with COVID-19 were admitted during the study period. Of these patients, 24 patients were diagnosed as pneumothorax or pneumomediastinum. When comparing the characteristics of both groups, body mass index (BMI) was significantly lower in the case group (22.8 kg/m^2^ and 24.7 kg/m^2^; *P* = 0.048). BMI was statistically significant risk factor for barotrauma in univariate conditional logistic regression analysis (Odds ratio (OR), 0.85; Confidence interval (CI), 0.72-0.996; *P* = 0.044) but not in multivariate analysis. For the patients with invasive mechanical ventilation, the period from symptom onset to intubation was longer in the case-patients (13 and 9.5 days; *P* = 0.032). Univariate conditional logistic regression analysis showed the statistical significance of the period from symptom onset to intubation (OR, 1.14; CI, 1.006-1.293; *P* = 0.041).

**Figure:**
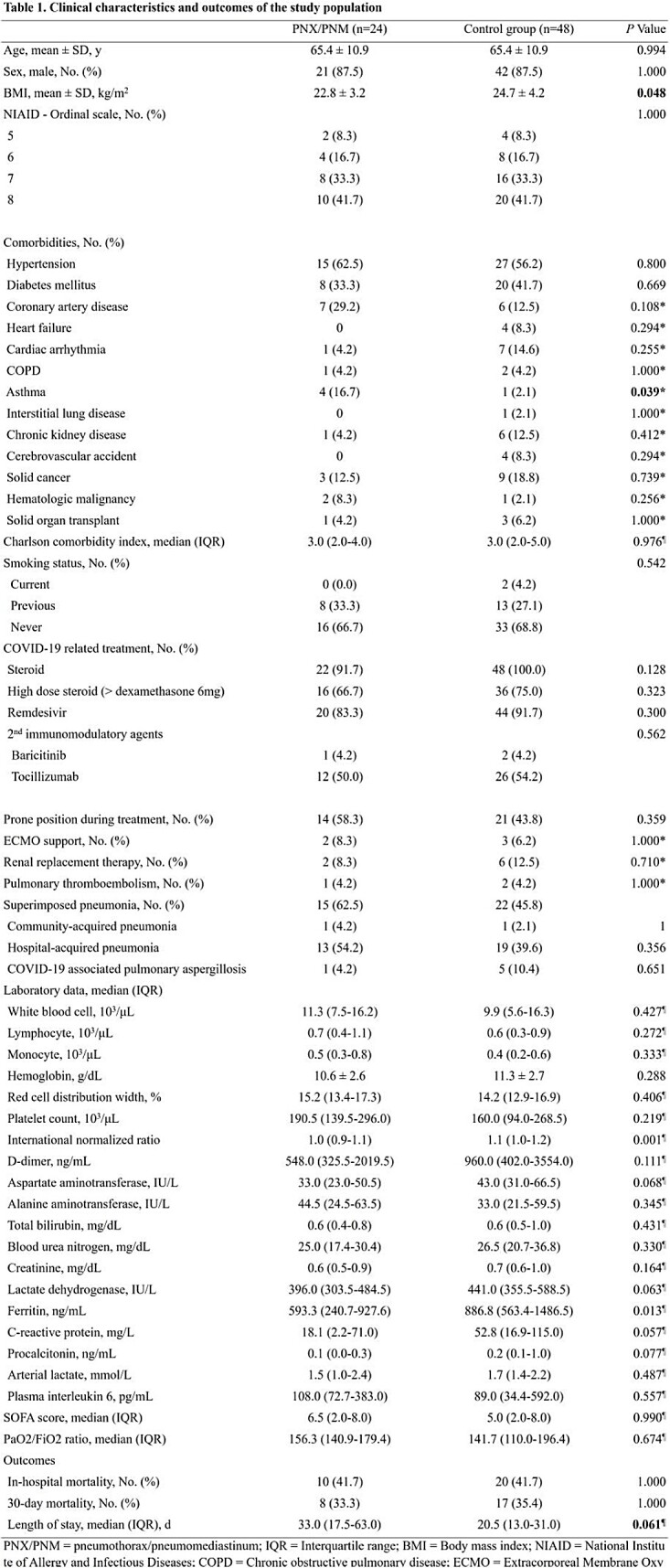

**Figure:**
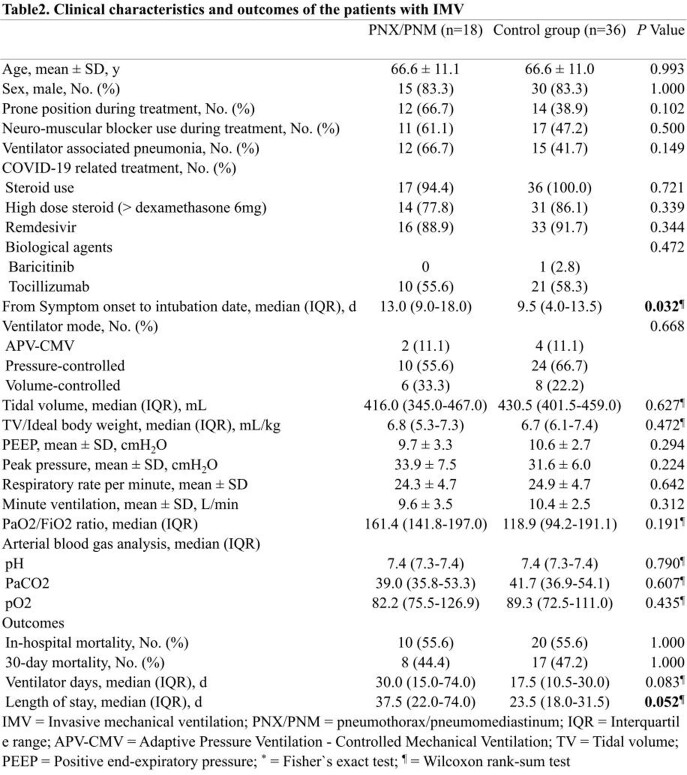

**Figure:**
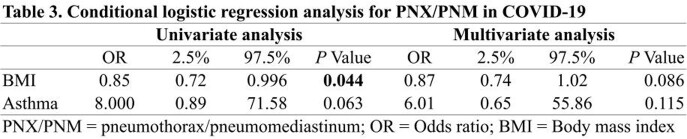

**Conclusion:**

In this case-control study with age, gender, severity matching, lower BMI was associated with the pneumothorax in COVID-19, and delayed application of invasive mechanical ventilation might contribute to this complication.

**Disclosures:**

**All Authors**: No reported disclosures.

